# Efficacy and Safety of SA001 in Patients with Primary Sjögren’s Syndrome: A Randomized, Double-Blind, Placebo-Controlled Trial

**DOI:** 10.3390/ph19010189

**Published:** 2026-01-22

**Authors:** Jaewon Park, Kyoung Yul Seo, Hyunmin Ahn, Yearim Shin, Ikhyun Jun, Tae-im Kim, Bum Kyu Shin, Da-Young Yoon, Soo-Min Lee

**Affiliations:** 1 Research Center, Samjin Pharmaceutical Co., Ltd., 90, Magokjungang 10-ro, Gangseo-gu, Seoul 07794, Republic of Korea; jwpark130@samjinpharm.co.kr (J.P.);; 2 Department of Ophthalmology, Yonsei University College of Medicine, 50-1 Yonsei-ro, Seodaemun-gu, Seoul 03722, Republic of Korea; seoky@yuhs.ac; 3 Severance Hospital, Yonsei University College of Medicine, Seoul 03722, Republic of Korea; 4 Shine Bright Vision Eye Clinic, Seoul 06611, Republic of Korea

**Keywords:** primary Sjögren’s syndrome, sicca symptoms, dry eye, xerostomia, rebamipide, SA001, randomized controlled trial, Phase 2a, ocular surface, salivary flow

## Abstract

**Background/Objectives**: SA001, a mofetil-ester prodrug of rebamipide, was developed to enhance gastrointestinal absorption and systemic exposure, which was confirmed in a prior Phase 1 study. Given the limited efficacy of current symptomatic therapies for primary Sjögren’s syndrome (pSS), this trial aimed to assess whether the improved bioavailability of SA001 could translate into clinical benefits. **Methods:** This multicenter, randomized, double-blind, placebo-controlled Phase 2a study enrolled adults who met the 2016 ACR–EULAR criteria for pSS. The participants were randomly assigned to one of four groups: SA001 at 360, 720, or 1080 mg/day (administered twice daily for 8 weeks) or placebo. Exploratory ocular assessments included tear break-up time, ocular surface staining, the Schirmer test, and the Standard Patient Evaluation of Eye Dryness. Oral endpoints included unstimulated whole salivary flow and the Xerostomia Inventory. Anti-SSA(Ro) antibodies were assessed both quantitatively and qualitatively. Safety evaluations comprised adverse events (AEs), ophthalmic examinations, laboratory tests, and vital signs. The efficacy outcomes were exploratory, and this study was not powered to formally test efficacy hypotheses. **Results**: Twenty-eight women (mean age 58.54 ± 9.29 years; range 41–75 years) were enrolled in this study and randomly assigned to one of the study groups. SA001 showed no statistically significant improvements versus placebo in ocular or oral endpoints, and no consistent dose–response relationship was observed. The anti-SSA(Ro) findings did not differ meaningfully across the groups. SA001 was generally well-tolerated, with infrequent, mostly mild-to-moderate AEs; however, one serious AE occurred in the placebo group. No clinically relevant ophthalmic or laboratory safety signals were detected. **Conclusions**: Despite the fact that markedly increased systemic exposure has been demonstrated previously, SA001 did not improve the dryness outcomes in pSS. These findings suggest that systemic exposure alone may be insufficient in established glandular disease and highlight the need for tissue-exposure-driven strategies and biomarker-informed patient selection in future studies. Predefined primary efficacy endpoints and objective, gland-proximal measures of target engagement (e.g., standardized salivary gland ultrasonography and salivary or tear fluid biomarker assessments) may help to better interpret local pharmacodynamic activity and the likelihood of a clinically meaningful benefit.

## 1. Introduction

Sjögren’s syndrome (SjS) is a chronic systemic autoimmune disorder that primarily affects the exocrine glands, especially the lacrimal and salivary glands [1]. Progressive lymphocytic infiltration and proinflammatory cytokine release drive destruction of glandular tissue and impaired secretory function [2,3,4]. SjS is classified as either primary or secondary. In primary Sjögren’s syndrome (pSS), current therapies are largely symptomatic [5], including topical tear substitutes, muscarinic receptor agonists, and immunomodulatory agents [6,7,8,9,10]. However, these treatments are limited by modest efficacy, a short duration of action, systemic adverse effects, and adherence challenges [5]. Importantly, these approaches do not directly address the underlying immunologic mechanisms responsible for progressive glandular dysfunction [11,12].

Rebamipide, originally developed as a gastric mucosal protective agent, has demonstrated secretory and anti-inflammatory properties; subsequently, it has been approved as a 2% ophthalmic suspension for the treatment of dry eye disease [13,14,15]. However, its poor oral bioavailability (~10%) has limited its use as a systemic therapy [16]. To address this, SA001 was developed as a mofetil-ester prodrug to enhance gastrointestinal absorption and systemic exposure. In a first-in-human Phase 1 trial (NCT02470286, NCT05303961), SA001 was rapidly absorbed and converted to rebamipide, producing dose-proportional pharmacokinetics and markedly higher systemic exposure compared with conventional rebamipide tablets while also demonstrating a favorable safety and tolerability profile [17]. Building on these findings, the dose range for the subsequent evaluation was prespecified from the multiple ascending dose (MAD) study: daily doses of 360, 720, and 1080 mg were administered over 16 days— twice daily (BID) for the first 8 days, followed by three times daily (TID) for the next 8 days—under both fasted and fed conditions. Across this range, exposure increased dose-proportionally, all regimens were well-tolerated, and no clinically significant safety concerns were identified [17], supporting the selection of 360–1080 mg/day for the next study.

Accordingly, we conducted a Phase 2a exploratory study in patients with pSS to evaluate the clinical efficacy and safety of SA001 and inform endpoint selection for future confirmatory trials. This report specifically examines whether the favorable bioavailability observed in the Phase 1 study translates into meaningful improvements in signs and symptoms in patients and explores the relationship between systemic exposure and clinical outcomes, considering the possibility that inadequate tissue-level drug delivery could account for limited efficacy.

## 2. Results

### 2.1. Participant Disposition

A total of 38 patients with primary Sjögren’s syndrome were assessed for eligibility, of whom 28 met the eligibility criteria and were randomly assigned to one of the four study groups. Participant recruitment was conducted between May 2020 and January 2022, and all participants were monitored for efficacy outcomes during the 8-week treatment period and for safety outcomes through a 2-week post-treatment follow-up.

In Group 1, three participants decided not to partake in the study after randomization: two withdrew their consent, and one was excluded due to a violation of eligibility criteria. In Group 2, one participant was excluded after randomization because of a violation of eligibility criteria. No participants were excluded from Group 3 or the placebo group.

**Figure 1:**
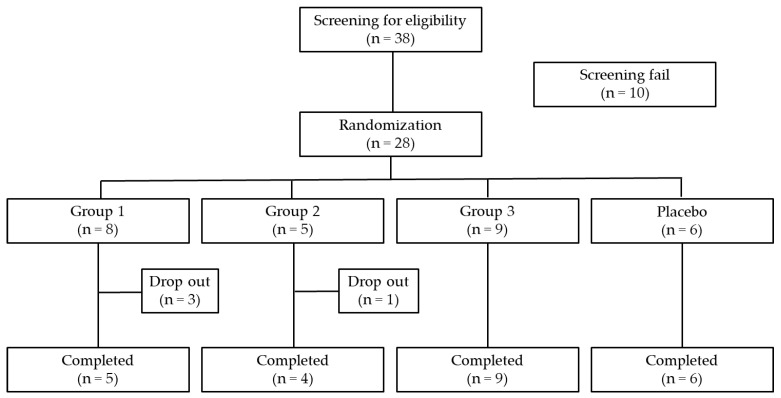
Flow diagram of participant disposition. Group 1: SA001 360 mg/day (180 mg twice daily); Group 2: SA001 720 mg/day (360 mg twice daily); Group 3: SA001 1080 mg/day (540 mg twice daily); placebo group.

All of the remaining randomized participants received the assigned intervention and were included in the full analysis set. Additional exclusions from the per-protocol set occurred due to post hoc eligibility violations (one participant in Group 2) and prohibited concomitant medication use (one participant each in Groups 1 and 3). The flow of participants through this study is shown in Figure 1.

### 2.2. Baseline Characteristics

Twenty-eight patients were enrolled in this study between 29 May 2020 and 7 January 2022. Summary statistics for the patients’ demographic characteristics, such as age, sex, weight, and height, are presented in Table 1 for each allocation group. All participants were women; the mean age in each group ranged from 53 to 66 years.

During the screening period, the medical history of the participants was assessed in 27 out of 28 participants (96.43%), with a total of 112 cases recorded. The most frequently reported medical histories according to System Organ Class (SOC) classification were eye disorders (40 cases, 35.72%), musculoskeletal and connective tissue disorders (18 cases, 16.08%), and surgical and medical procedures (13 cases, 11.61%). For Preferred Term (PT) classification, the most commonly reported conditions were dry eye (18 cases, 16.08%), punctal plug insertion (8 cases, 7.15%), and osteoporosis (7 cases, 6.25%). Detailed medical history of the patients is provided in Supplementary Table S1.

### 2.3. Efficacy

Efficacy outcomes were assessed as exploratory endpoints; therefore, the results are presented by clinical domain (ocular signs, oral function, and serologic biomarkers) to describe potential dose-related patterns rather than to confirm clinical efficacy.

During the study, concomitant use of carboxymethylcellulose (CMC)-containing artificial tears was permitted as rescue medication across all treatment groups.

#### 2.3.1. Dry Eye

##### TBUT

**Figure 2:**
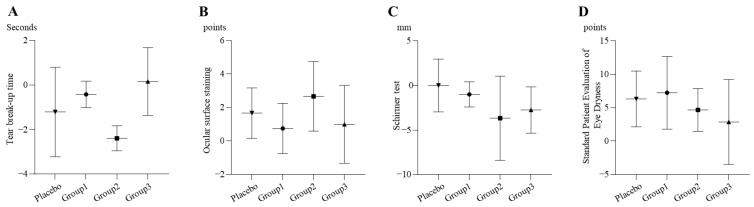
Changes in dry eye signs and symptoms from baseline to week 8 in the per-protocol set; change = baseline − week 8. Bars indicate mean ± SD (Group 1 n = 4, Group 2 n = 3, Group 3 n = 8, and placebo n = 6). (**A**) Tear break-up time (TBUT): Negative values indicate improvement (increase in TBUT). No statistically significant differences were observed among the groups (*p* = 0.1006), and no dose-dependent trend was evident across the active treatment groups. (**B**) Ocular surface staining (OSS): Positive values indicate improvement (reduced staining). No statistically significant differences were observed among the groups (*p* = 0.5549), and no dose-dependent trend was evident across the active treatment groups. (**C**) Schirmer test: Negative values indicate improvement (increase in tear production). No statistically significant differences were observed among the groups (*p* = 0.2309), and no dose-dependent trend was evident across the active treatment groups. (**D**) Standard Patient Evaluation of Eye Dryness (SPEED): Positive values indicate improvement (decrease in SPEED score). No statistically significant differences were observed among the groups (*p* = 0.5132), and no dose-dependent trend was evident across the active treatment groups.

From the baseline to week 8, mean TBUT increased by 0.42 ± 0.59 s in Group 1, 2.39 ± 0.56 s in Group 2, and 1.21 ± 2.01 s in the placebo group, whereas Group 3 showed a decrease of 0.16 ± 1.53 s (*p* = 0.1006) (see Figure 2A).

##### OSS

From the baseline to week 8, the OSS scores decreased by 0.75 ± 1.50, 2.67 ± 2.08, 1.00 ± 2.33, and 1.67 ± 1.51 points in Groups 1, 2, and 3 and the placebo group, respectively (*p* = 0.5549) (see Figure 2B).

##### Schirmer Test

From the baseline to week 8, the Schirmer test values increased by 1.00 ± 1.41 mm, 3.67 ± 4.73 mm, and 2.75 ± 2.60 mm in Groups 1, 2, and 3, respectively. In contrast, no difference was observed in the placebo group (*p* = 0.2309) (see Figure 2C).

##### SPEED

From the baseline to week 8, the SPEED scores decreased by 7.25 ± 5.44, 4.67 ± 3.21, 2.88 ± 6.36, and 6.33 ± 4.18 points in Groups 1, 2, and 3 and the placebo group, respectively (*p* = 0.5132) (see Figure 2D).

Overall, there was no evidence of a dose-dependent effect in any of the treatment groups.

#### 2.3.2. Dry Mouth

##### The Unstimulated Whole Salivary Flow Rate

**Figure 3:**
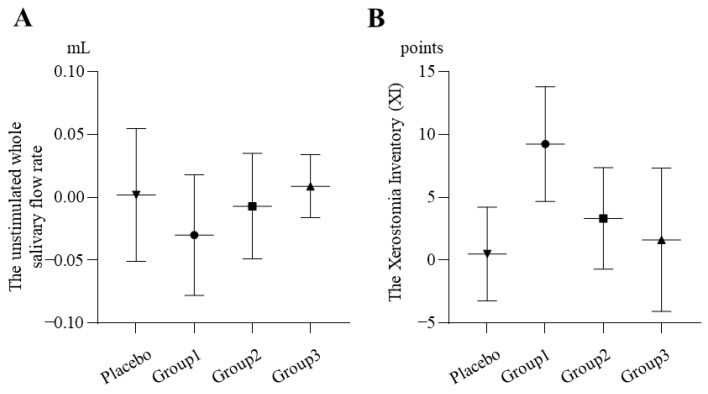
Changes in dry mouth signs and symptoms from baseline to week 8 in the per-protocol set; change = baseline − week 8. Bars indicate mean ± SD (Group 1 n = 4, Group 2 n = 3, Group 3 n = 8, and placebo n = 6). (**A**) The unstimulated whole salivary flow rate: Negative values indicate improvement (increase in salivary flow rate). No statistically significant differences were observed among the groups (*p* = 0.5017), and no dose-dependent trend was evident across the active treatment groups. (**B**) The Xerostomia Inventory (XI): Positive values indicate improvement (decrease in the XI score). No statistically significant differences were observed among the groups (*p* = 0.0586), and no dose-dependent trend was evident across the active treatment groups.

From the baseline to week 8, the unstimulated salivary flow rate decreased by 0.009 ± 0.025 mL and 0.002 ± 0.053 mL in Group 3 and the placebo group, respectively. In contrast, an increase of 0.030 ± 0.048 mL and 0.007 ± 0.042 mL was observed in Group 1 and Group 2, respectively (*p* = 0.5017) (see Figure 3A).

##### XI Questionnaire

From the baseline to week 8, the XI scores decreased by 9.25 ± 4.57, 3.33 ± 4.04, 1.63 ± 5.71, and 0.50 ± 3.73 points in Groups 1, 2, and 3 and the placebo group, respectively (*p* = 0.0586) (see Figure 3B).

Overall, there was no evidence of a dose-dependent effect in any of the treatment groups.

#### 2.3.3. Biomarkers

The quantitative assessment of SSA (Ro) showed an increase in treatment Group 1 and Group 3, with changes of 0.03 ± 0.22 and 0.04 ± 0.12 from the screening period to week 8, respectively. In contrast, Group 2 and the placebo group showed decreases of 0.04 ± 0.08 and 0.07 ± 0.20, respectively (*p* = 0.6240) (see Table 2).

For the qualitative assessment, no changes were observed in any of the treatment groups from the baseline to week 8, while one participant in the placebo group changed from positive to negative.

Additionally, no statistically significant changes or between-group differences were observed for SSB (La), ANA, RF, total IgG, or IgA; detailed results are provided in Supplementary Table S2.

### 2.4. Safety

All participants received the assigned investigational product according to the study protocol. Study medication was administered orally, and no deviations in the dosing schedule or treatment administration were identified.

Adverse events (AEs) occurred in 5 of 22 subjects (22.7%) receiving SA001 and in 1 of 6 subjects (16.7%) receiving placebo, with a total of six events. Three were Grade 1 (eye pain [two cases], dyspnea) and three were Grade 2 (aggravation of renal calculi, adhesive capsulitis of the shoulder, sensorineural hearing loss). Five events were considered to be unrelated, and one was unlikely to be related to the investigational drug. All AEs were resolved or were resolving at study completion, and the single serious AE (aggravation of renal calculi) occurred in the placebo group. No clinically meaningful changes were observed in visual acuity, intraocular pressure, vital signs, or laboratory parameters; a group difference in urine pH was noted, but it remained within the normal range. A detailed summary of the AEs is presented in Table 3.

## 3. Discussion

This exploratory Phase 2a study evaluated whether the marked increases in systemic exposure to rebamipide achieved with the oral prodrug SA001 would translate into improvements in signs and symptoms in pSS. Despite the favorable pharmacokinetics and tolerability demonstrated in the Phase 1 study [17], the efficacy outcomes did not show a clear separation from the placebo across ocular or oral dryness measures, and no consistent dose-related pattern was evident across the active treatment arms. Given the exploratory design and limited cohort size, these findings should be interpreted as hypothesis-generating rather than confirmatory. Consistent with this intent, this study was not powered to formally evaluate efficacy, and the small per-group sample sizes—as few as n = 3–4 in the per-protocol set—substantially limit statistical inference and increase sensitivity to individual outliers (see Supplementary Figures S1–S3 for details).

It should also be noted that all the participants enrolled in this study were women, reflecting the marked female predominance of primary Sjögren’s syndrome [3]; as a result, there may be potential differences in treatment response by sex, potentially limiting the generalizability of the findings to male patients, but assessing these possible differences was outside the scope of the present analysis. In addition, multiple ocular and oral endpoints were assessed without a single predefined primary efficacy endpoint, an approach appropriate for exploratory signal detection but one that necessitates cautious interpretation in light of multiplicity. Taken together, these design features frame the present results as an initial evaluation of potential clinical signals rather than definitive evidence of efficacy.

Within this exploratory framework, the overall pattern of the findings raises the possibility of a pharmacokinetic–pharmacodynamic disconnect. pSS is driven by chronic lymphocytic infiltration and progressive damage to lacrimal and salivary glands; reversing dryness endpoints over short intervals with systemic agents has often been challenging across trials [12,18,19], and this is reflected in the contemporary European Alliance of Associations for Rheumatology (EULAR) management guidance, which emphasizes predominantly symptomatic care and the difficulty of demonstrating a change in dryness domains [12]. This disease biology may help to explain why larger, well-controlled studies of systemic immunomodulators have not consistently delivered durable improvements in sicca outcomes [18,19].

From a pharmacologic standpoint, this lack of efficacy may reflect insufficient drug delivery to the relevant exocrine tissues despite markedly improved systemic exposure with SA001. Rebamipide itself shows extensive plasma protein binding (~98%) [20] and a tendency for preferential retention in gastrointestinal mucosa [21], properties that could bias distribution away from salivary and lacrimal glands. Although the prodrug strategy or formulation improvement successfully increased plasma AUC and Cmax [17,22,23], these systemic gains may not have translated into adequate concentrations at glandular sites of disease activity. Such tissue-distribution disconnect offers a plausible mechanistic explanation for the absence of a clinical benefit despite higher systemic exposure.

Methodological considerations may have further contributed to the observed limitations. Commonly employed ophthalmic endpoints such as TBUT, the Schirmer test, and OSS, as well as patient-reported symptom scales, are recognized to exhibit substantial intra- and inter-visit variability and to be susceptible to placebo effects, particularly in short-duration or small-sample trials [24,25,26,27,28,29,30]. TBUT and the Schirmer test, in particular, have been reported to possess high measurement variability and limited reproducibility [24,26,27], thereby reducing the sensitivity to detect modest treatment effects without adequately powered sample sizes or extended treatment durations. In the present study, the 8-week treatment period and limited cohort size likely reduced statistical power; however, the absence of a consistent dose–response relationship across multiple endpoints, coupled with the influence of single-subject outliers (see Supplementary Figures S1–S3 for details), makes it less likely that the negative findings can be explained solely by type 2 error.

Against this methodological backdrop, the borderline trend observed in the XI (*p* = 0.0586) should be interpreted cautiously, given the limited sample size and multiplicity of the exploratory endpoints; nonetheless, this observation may inform endpoint selection for future, adequately powered studies incorporating objective or biomarker-linked measures.

Exploratory analyses of serologic biomarkers yielded consistent results with the clinical efficacy findings. Modest numerical reductions in total IgA and IgG concentrations were observed in the active treatment groups relative to the placebo group; however, the magnitude of these changes was modest and did not allow for clear clinical interpretation. No meaningful alterations were detected in the anti-SSA/Ro, anti-SSB/La, ANA, or rheumatoid factor levels. Previous studies have identified RF-IgA as a sensitive and specific serologic marker in pSS, showing strong associations with anti-SSA/SSB positivity and immunologic activity [31]. However, RF-IgA was not separately assessed in this study; instead, only total RF and total IgA levels were measured. The absence of RF-IgA assessment represents a limitation, as more sensitive immunologic biomarkers may better capture disease activity and pharmacodynamic effects in pSS. Consequently, over the 8-week treatment period, we did not detect a discernible pharmacodynamic signal.

The present findings are generally consistent with a prior randomized, placebo-controlled evaluation of oral rebamipide in patients with pSS-associated xerostomia, which demonstrated modest numerical advantages over the placebo at interim time points without statistically significant between-group differences at the final assessment [32]. This similarity in temporal response patterns suggests the lack of a durable, clinically meaningful benefit across studies. Notably, in pSS, interim improvements in salivary secretion and symptoms were statistically significant but not sustained, which the authors attributed to subjective endpoint limitations, the short treatment duration, and the need for longer-term administration [33]. These factors may likewise underlie the limitations observed in the present study.

In summary, this exploratory Phase 2a study did not demonstrate a clinically meaningful improvement in sicca manifestations of pSS with SA001 despite achieving markedly increased systemic exposure and maintaining a favorable safety profile [17]. Taken together with prior randomized experience using oral rebamipide, all evidence supports the interpretation that simply augmenting plasma exposure is unlikely to overcome the biological and methodological barriers to changing dryness endpoints over short timeframes. These results therefore refine—rather than end—our development hypothesis: for diseases characterized by site-specific pathology in exocrine glands, systemic pharmacokinetics alone may be an inadequate surrogate for therapeutic effect.

Looking ahead, the further development of SA001 or related approaches should be contingent upon prior demonstration of target-tissue delivery and pharmacodynamic engagement. In practical terms, this entails the following: (i) adopting tissue exposure-centric strategies—such as formulations or dosing regimens that enrich drug levels within salivary and lacrimal glands, or combined local–systemic administration [12,14,34]; (ii) enriching study populations for patients with residual glandular function, who may be more likely to respond [12,35,36,37]; (iii) extending treatment duration and ensuring adequate statistical power to address the limitations of short, small-sample trials [24,38]; and (iv) incorporating mechanistic, tissue-proximal endpoints—including standardized salivary gland ultrasonography and salivary or tear fluid biomarkers—to more directly capture gland-level structural or functional changes associated with the therapeutic intervention [39,40,41,42]. In addition, (v) prospective exposure–response and pharmacokinetics–pharmacodynamics modeling, including physiologically based pharmacokinetic approaches, should be considered to quantitatively link systemic exposure with tissue-level pharmacologic responses and to inform rational dose selection and predefined go/no-go criteria. Pending such evidence, the favorable tolerability observed in this study suggests a safety margin within which focused, mechanism-confirming investigations can be pursued prior to the performance of large-scale efficacy trials.

## 4. Materials and Methods

### 4.1. Study Design

This multicenter, double-blind, randomized Phase 2a study was designed as an exploratory trial to evaluate the safety and tolerability of SA001, with efficacy outcomes assessed on an exploratory basis, in patients with pSS presenting with dry eye and dry mouth symptoms. This study was approved by the Korean Ministry of Food and Drug Safety (MFDS) prior to study initiation (IND approval No. 30529, approved on 6 November 2019). Study documents were reviewed by the Institutional Review Board (IRB) of Yonsei Severance Hospital, Seoul ST. Mary’s Hospital, Inha University Hospital and Dankook University Hospital. The clinical trials were conducted in accordance with the Declaration of Helsinki and Korean Good Clinical Practice (KGCP) (ClinicalTrials.gov identifier: NCT05269810).

The study included four parallel arms: three dose levels of SA001 (360 mg/day [180 mg BID], 720 mg/day [180 mg × 2 BID], and 1080 mg/day [180 mg × 3 BID]) and one placebo arm. Group 1 received one tablet of SA001 180 mg twice daily (360 mg), Group 2 received two tablets of SA001 180 mg twice daily (720 mg), and Group 3 received three tablets of SA001 180 mg twice daily (1080 mg).

Participants who voluntarily agreed to participate underwent screening assessments according to the clinical trial protocol. After screening, eligible participants entered a one- to two-week run-in period, during which they used artificial tears containing carboxymethylcellulose (CMC) as needed, applied to the affected eye(s) according to the prescribed dosage and frequency.

After the run-in period, only participants who met the final inclusion and exclusion criteria were randomized in a double-blind manner to one of the four groups. The random allocation sequence was generated independently by a statistician, who was not otherwise involved in the conduct of the trial, using a computer-generated randomization method, and the randomization list was provided only to the personnel responsible for investigational product packaging. The participants, investigators, and study staff, including outcome assessors, were blinded to treatment allocation throughout the study. Blinding was maintained using SA001 and placebo tablets that were identical in appearance, packaging, and dosing schedule.

The maximum treatment duration was eight weeks, during which concomitant use of CMC-containing artificial tears was permitted. A two-week follow-up period was implemented after the treatment phase to monitor participant safety.

### 4.2. Participants and Eligibility

Eligible patients were adults aged 19–80 years who met the 2016 ACR–EULAR classification criteria for pSS [43]. Key exclusion criteria included secondary Sjögren’s syndrome, significant ocular comorbidities, and other systemic autoimmune diseases unrelated to SjS. A total of 28 participants were enrolled and randomly assigned to one of the four groups. Detailed inclusion and exclusion criteria are provided in Supplementary Table S3.

### 4.3. Efficacy Assessments

Efficacy assessments were exploratory and encompassed multiple ocular and oral domains to screen for potential signals of clinical activity in pSS. Accordingly, exploratory efficacy endpoints in the study included changes from baseline in both subjective and objective measures of ocular and oral dryness. Ocular assessments were performed at weeks 4 and 8 and included tear break-up time (TBUT), ocular surface staining (OSS) score, the Schirmer test, and the Standard Patient Evaluation of Eye Dryness (SPEED) questionnaire. Oral dryness was evaluated at week 8 using the unstimulated whole salivary flow rate and the Xerostomia Inventory (XI) score. Additionally, changes in anti-SSA (Ro) antibody levels were analyzed at week 8 as an exploratory immunologic endpoint. Further exploratory biomarker analyses included quantitative and qualitative assessment of anti-SSB (La) antibodies, qualitative assessment of antinuclear antibodies (ANA), and quantification of rheumatoid factor (RF), immunoglobulin G (IgG), and immunoglobulin A (IgA) levels.

### 4.4. Safety Assessments

The study assessments included visual acuity, intraocular pressure, laboratory analysis, and vital signs. Also, patients were assessed for AEs at each study visit. Visual acuity, intraocular pressure, and laboratory safety assessments were conducted at visit 1 (screening) and week 8, including hematology, coagulation, chemistry, and urinalysis. All adverse events were coded according to the Medical Dictionary for Regulatory Activities (MedDRA) and assessed by the investigators for severity and their relationship to the investigational product.

### 4.5. Statistical Analysis

In this study, summary statistics were presented for all collected information for each treatment group and placebo group, as well as the overall study population. Where necessary, 95% confidence intervals were provided. Demographic information, disease-related information, and other relevant data were also summarized with statistical measures for each treatment group and placebo group, as well as the overall study population.

Efficacy analyses were conducted in the full analysis set and the per-protocol set according to assigned treatment groups, whereas safety analyses included all participants who received at least one dose of the investigational product.

To explore the dose–response relationship among treatment groups, it was planned to fit (generalized) linear models to examine various dose–response patterns such as linear, step, umbrella, convex, and concave relationships, and evaluate the appropriate dose–response relationship. The efficacy evaluation results for all study participants were graphically represented and comprehensively reviewed. The presence of any anomalies for individual participants was assessed, their clinical significance was documented, and their potential relationship with the investigational product was examined.

Comparisons between treatment groups and the placebo group were based on descriptive summaries and exploratory modeling rather than formal hypothesis-testing. Missing data were not imputed, and analyses were performed using available data only. Safety outcomes were summarized descriptively by treatment group and the placebo group.

No formal sample size calculation for efficacy was performed, as this study was exploratory in nature. Accordingly, statistical analyses of efficacy outcomes were descriptive and intended to support signal detection rather than confirmatory inference. *p*-values, where reported, were interpreted descriptively in the context of the small size and multiple exploratory endpoints.

## 5. Conclusions

In this exploratory Phase 2a study on pSS, SA001 did not produce clinically meaningful improvements in ocular or oral dryness outcomes versus placebo, and no consistent dose–response relationship was observed. Although the Phase 1 study established that SA001 markedly increases systemic exposure to rebamipide and is well-tolerated [17], these pharmacokinetic gains did not translate into efficacy over the 8-week trial. Taken together with prior randomized studies on oral rebamipide [32], the evidence suggests that in gland-centric diseases, plasma exposure alone is not an adequate surrogate for therapeutic effect.

While tolerability was favorable, the present findings indicate that large-scale efficacy trials may be premature in the absence of convincing evidence for target-tissue delivery and pharmacodynamic engagement. Further development should, therefore, focus on mechanism-confirming investigations with predefined primary efficacy endpoints. Such studies should incorporate an explicit assessment of target-gland engagement using objective, gland-proximal measures, including imaging-based evaluation with standardized salivary gland ultrasonography and gland-relevant biomarker assessments in saliva and/or tear fluid [39,40,41,42]. This approach would help clarify whether tissue-directed strategies can meaningfully translate pharmacokinetic gains into clinical benefit.

## Figures and Tables

**Table 1:** Baseline characteristics.

		Total		Group 1		Group 2		Group 3		Placebo		*p*-Value
		N = 28		N = 8		N = 5		N = 9		N = 6		
		N	%	N	%	N	%	N	%	N	%	
Sex (N, %)	Male	0	0.00	0	0.00	0	0.00	0	0.00	0	0.00	-
	Female	28	100.00	8	100.00	5	100.00	9	100.00	6	100.00	
Age (years)	Mean	58.54		56.13		66.00		60.00		53.33		0.1113
	SD	9.29		11.47		6.96		4.09		10.71		
	Median	59.00		56.00		66.00		59.00		53.50		
	Min	41.00		42.00		56.00		53.00		41.00		
	Max	75.00		75.00		75.00		67.00		69.00		
Height (cm)	Mean	158.28		160.21		157.56		156.46		159.02		0.5278
	SD	5.30		4.74		7.47		3.94		6.12		
	Median	157.70		160.45		157.20		157.00		160.10		
	Min	150.00		154.20		150.20		150.00		150.20		
	Max	169.20		166.20		169.20		162.50		165.00		
Weight (kg)	Mean	55.08		60.79		49.60		55.66		51.15		0.1040
	SD	9.18		8.30		6.87		10.88		5.49		
	Median	52.65		59.55		51.80		51.40		50.25		
	Min	40.20		51.00		40.20		46.10		45.00		
	Max	76.10		74.60		58.00		76.10		60.50		
History of punctal occlusion or other treatments, procedures, or surgeries for symptom improvement	Yes	14	50.00	4	50.00	3	60.00	3	33.33	4	66.67	0.6233
	No	14	50.00	4	50.00	2	40.00	6	66.67	2	33.33	

Group 1: SA001 360 mg/day (180 mg twice daily); Group 2: SA001 720 mg/day (360 mg twice daily); Group 3: SA001 1080 mg/day (540 mg twice daily); placebo group. N = number of participants.

**Table 2:** Anti-SSA (Ro) antibody changes from visit 2 (baseline) to visit 4 (week 8) in each group.

		Group 1		Group 2		Group 3		Placebo		*p*-Value
		N = 4		N = 3		N = 8		N = 6		
		Mean	SD	Mean	SD	Mean	SD	Mean	SD	
Visit 2		1.44	0.04	1.41	0.15	1.29	0.04	1.42	0.23	0.2420
Visit 4		1.47	0.25	1.37	0.20	1.33	0.13	1.35	0.24	0.7281
Δ (Visit 2–Visit 4)		−0.03	0.22	0.04	0.08	−0.04	0.12	0.07	0.20	0.6240
		N	%	N	%	N	%	N	%	
Visit 2	Positive	4	100.00	3	100.00	8	100.00	6	100.00	-
	Negative	0	0.00	0	0.00	0	0.00	0	0.00	
Visit 4	Positive	4	100.00	3	100.00	8	100.00	5	83.33	0.6190
	Negative	0	0.00	0	0.00	0	0.00	1	16.67	

Group 1: SA001 360 mg/day (180 mg twice daily); Group 2: SA001 720 mg/day (360 mg twice daily); Group 3: SA001 1080 mg/day (540 mg twice daily); placebo group. N = number of participants.

**Table 3:** A summary of adverse events.

SOC/PT	Total		Group 1		Group 2		Group 3		Placebo	
	N = 28		N = 8		N = 5		N = 9		N = 6	
Adverse Events	N	%	N	%	N	%	N	%	N	%
Ear and labyrinth disorders	1	16.67	1	100.00	0	0.00	0	0.00	0	0.00
Deafness neurosensory	1	16.67	1	100.00	0	0.00	0	0.00	0	0.00
Eye disorders	2	33.33	0	0.00	0	0.00	2	100.00	0	0.00
Eye pain	2	33.33	0	0.00	0	0.00	2	100.00	0	0.00
Musculoskeletal and connective tissue disorders	1	16.67	0	0.00	1	50.00	0	0.00	0	0.00
Periarthritis	1	16.67	0	0.00	1	50.00	0	0.00	0	0.00
Renal and urinary disorders	1	16.67	0	0.00	0	0.00	0	0.00	1	100.00
Nephrolithiasis	1	16.67	0	0.00	0	0.00	0	0.00	1	100.00
Respiratory, thoracic. and mediastinal disorders	1	16.67	0	0.00	1	50.00	0	0.00	0	0.00
Dyspnea	1	16.67	0	0.00	1	50.00	0	0.00	0	0.00

Group 1: SA001 360 mg/day (180 mg twice daily); Group 2: SA001 720 mg/day (360 mg twice daily); Group 3: SA001 1080 mg/day (540 mg twice daily); placebo group. N = number of participants.

## Data Availability

The original contributions presented in this study are included in the article and Supplementary Material. Further inquiries can be directed to the corresponding author.

## Supplementary Materials

The following supporting information can be downloaded at https://www.mdpi.com/article/10.3390/ph19010189/s1. Table S1: Medical history; Table S2: Changes in exploratory biomarkers (SSB (La), ANA, RF, IgG, IgA) from visit 2 (baseline) to visit 4 (week 8) in each group; Table S3: Inclusion and exclusion criteria for SA001_04 Phase 2a; Figure S1: OSS scores at each time point and change from baseline to week 8 in treatment Group 2; Figure S2: Shirmer test at each time point and change from baseline to week 8 in treatment Group 2; Figure S3: The unstimulated whole salivary flow rate at each time point and change from baseline to week 8 in treatment Group 1.

## Abbreviations

The following abbreviations are used in this manuscript:

ACR	American College of Rheumatology
AE/AEs	Adverse Event(s)
ANA	Antinuclear Antibody
AUC	Area Under the (plasma) Concentration–Time Curve
BID	Twice Daily
CI	Confidence Interval(s)
CMC	Carboxymethylcellulose
C_max_	Maximum (plasma) Concentration
DEWS	Dry Eye Workshop
EULAR	European Alliance of Associations for Rheumatology
GCP	Good Clinical Practice
IgA	Immunoglobulin A
IgG	Immunoglobulin G
IRB	Institutional Review Board
KGCP	Korean Good Clinical Practice
MAD	Multiple Ascending Dose
MedDRA	Medical Dictionary for Regulatory Activities
MFDS	Ministry of Food and Drug Safety (Republic of Korea)
OSS	Ocular Surface Staining
pSS	Primary Sjögren’s Syndrome
PK	Pharmacokinetics
PT	Preferred Term (MedDRA term level)
RF	Rheumatoid Factor
RF-IgA	IgA Isotype of Rheumatoid Factor
SD	Standard Deviation
SjS	Sjögren’s Syndrome
SOC	System Organ Class
SPEED	Standard Patient Evaluation of Eye Dryness
SSA	Anti-Ro (SSA) Antibody
SSB	Anti-La (SSB) Antibody
TBUT	Tear Break-Up Time
TID	Three Times Daily
XI	Xerostomia Inventory
