# Supplementary material for: Efficacy and Safety of SA001 in Patients with Primary Sjögren’s Syndrome: A Randomized, Double-Blind, Placebo-Controlled Trial
Source: Pharmaceuticals (Basel). 2026 Jan 22;19(1):189. doi: 10.3390/ph19010189 (PMC12844686; doi:10.3390/ph19010189)
Supplement: Supplementary file 1 [file pharmaceuticals-19-00189-s001.zip › pharmaceuticals-4086383-supplementary.pdf]

**Table S1**	**Medical History**
**Table S2**	**Exploratory Biomarkers (SSB (La), ANA, RF, IgG, IgA) Changes from Visit 2 (Baseline) to Visit 4 (Week 8) in Each Group**
**Table S3**	**Inclusion and exclusion criteria for SA001_04 Phase 2a**
**Figure S1**	**OSS Scores at Each Time Point and Change from Baseline to Week 8 in Treatment Group 2**
**Figure S2**	**Shirmer test at Each Time Point and Change from Baseline to Week 8 in Treatment Group 2**
**Figure S3**	**The unstimulated whole salivary flow rate at Each Time Point and Change from Baseline to Week 8 in Treatment Group 1**

Supplementary Table S1. Medical History

	Total		Group 1		Group 2		Group 3		Placebo	
	N= 28		N= 8		N= 5		N= 9		N= 6	
	N	%	N	%	N	%	N	%	N	%
N	27	96.43	7	87.50	5	100.00	9	100.00	6	100.00
Cases	112		32		28		35		17	
SOC/PT										
**Blood and lymphatic system disorders**	**5**	**4.47**	**2**	**6.25**	**1**	**3.58**	**2**	**5.72**	**0**	**0.00**
Eosinophilia	1	0.90	1	3.13	0	0.00	0	0.00	0	0.00
Leukopenia	2	1.79	0	0.00	1	3.58	1	2.86	0	0.00
Thrombocytopenia	2	1.79	1	3.13	0	0.00	1	2.86	0	0.00
**Ear and labyrinth disorders**	**1**	**0.90**	**1**	**3.13**	**0**	**0.00**	**0**	**0.00**	**0**	**0.00**
Tinnitus	1	0.90	1	3.13	0	0.00	0	0.00	0	0.00
**Endocrine disorders**	**2**	**1.79**	**1**	**3.13**	**0**	**0.00**	**0**	**0.00**	**1**	**5.89**
Hypothyroidism	2	1.79	1	3.13	0	0.00	0	0.00	1	5.89
**Eye disorders**	**40**	**35.72**	**10**	**31.25**	**11**	**39.29**	**12**	**34.29**	**7**	**41.18**
Astigmatism	2	1.79	1	3.13	0	0.00	0	0.00	1	5.89
Blepharitis	3	2.68	1	3.13	0	0.00	2	5.72	0	0.00
Borderline glaucoma	2	1.79	1	3.13	0	0.00	1	2.86	0	0.00
Cataract	2	1.79	0	0.00	2	7.15	0	0.00	0	0.00
Corneal erosion	3	2.68	1	3.13	1	3.58	0	0.00	1	5.89
Dry eye	18	16.08	4	12.50	5	17.86	6	17.15	3	17.65
Entropion	1	0.90	1	3.13	0	0.00	0	0.00	0	0.00
Keratitis	2	1.79	0	0.00	1	3.58	1	2.86	0	0.00
Meibomian gland dysfunction	1	0.90	0	0.00	0	0.00	1	2.86	0	0.00
Punctate keratitis	3	2.68	1	3.13	1	3.58	0	0.00	1	5.89
Retinal disorder	1	0.90	0	0.00	0	0.00	0	0.00	1	5.89
Retinal drusen	1	0.90	0	0.00	1	3.58	0	0.00	0	0.00
Vitreous floaters	1	0.90	0	0.00	0	0.00	1	2.86	0	0.00
**Gastrointestinal disorders**	**4**	**3.58**	**1**	**3.13**	**1**	**3.58**	**2**	**5.72**	**0**	**0.00**
Dry mouth	2	1.79	0	0.00	1	3.58	1	2.86	0	0.00
Irritable bowel syndrome	1	0.90	1	3.13	0	0.00	0	0.00	0	0.00
Oesophagitis	1	0.90	0	0.00	0	0.00	1	2.86	0	0.00
**Hepatobiliary disorders**	**1**	**0.90**	**0**	**0.00**	**1**	**3.58**	**0**	**0.00**	**0**	**0.00**
Hepatic cirrhosis	1	0.90	0	0.00	1	3.58	0	0.00	0	0.00
**Infections and infestations**	**9**	**8.04**	**2**	**6.25**	**4**	**14.29**	**1**	**2.86**	**2**	**11.77**
Abscess of eyelid	1	0.90	1	3.13	0	0.00	0	0.00	0	0.00
Chronic hepatitis B	1	0.90	0	0.00	1	3.58	0	0.00	0	0.00
Cystitis	1	0.90	0	0.00	0	0.00	0	0.00	1	5.89
Herpes zoster	1	0.90	0	0.00	0	0.00	0	0.00	1	5.89

Oral candidiasis	1	0.90	0	0.00	1	3.58	0	0.00	0	0.00
Periodontitis	3	2.68	0	0.00	2	7.15	1	2.86	0	0.00
Upper respiratory tract infection	1	0.90	1	3.13	0	0.00	0	0.00	0	0.00
**Musculoskeletal and connective tissue disorders**	**18**	**16.08**	**3**	**9.38**	**5**	**17.86**	**8**	**22.86**	**2**	**11.77**
Arthralgia	2	1.79	0	0.00	0	0.00	2	5.72	0	0.00
Cervical spinal stenosis	1	0.90	0	0.00	1	3.58	0	0.00	0	0.00
Lumbar spinal stenosis	1	0.90	0	0.00	0	0.00	1	2.86	0	0.00
Osteoarthritis	2	1.79	0	0.00	0	0.00	1	2.86	1	5.89
Osteopenia	3	2.68	1	3.13	0	0.00	1	2.86	1	5.89
Osteoporosis	7	6.25	2	6.25	3	10.72	2	5.72	0	0.00
Spinal osteoarthritis	1	0.90	0	0.00	1	3.58	0	0.00	0	0.00
Trigger finger	1	0.90	0	0.00	0	0.00	1	2.86	0	0.00
**Neoplasms benign, malignant and unspecified (incl cysts and polyps)**	**1**	**0.90**	**1**	**3.13**	**0**	**0.00**	**0**	**0.00**	**0**	**0.00**
Benign breast neoplasm	1	0.90	1	3.13	0	0.00	0	0.00	0	0.00
**Nervous system disorders**	**3**	**2.68**	**0**	**0.00**	**0**	**0.00**	**3**	**8.58**	**0**	**0.00**
Headache	1	0.90	0	0.00	0	0.00	1	2.86	0	0.00
Paraesthesia	1	0.90	0	0.00	0	0.00	1	2.86	0	0.00
Spinal claudication	1	0.90	0	0.00	0	0.00	1	2.86	0	0.00
**Renal and urinary disorders**	**4**	**3.58**	**3**	**9.38**	**0**	**0.00**	**0**	**0.00**	**1**	**5.89**
Chronic kidney disease	1	0.90	1	3.13	0	0.00	0	0.00	0	0.00
Nephrocalcinosis	1	0.90	1	3.13	0	0.00	0	0.00	0	0.00
Nephrolithiasis	1	0.90	0	0.00	0	0.00	0	0.00	1	5.89
Neurogenic bladder	1	0.90	1	3.13	0	0.00	0	0.00	0	0.00
**Respiratory, thoracic and mediastinal disorders**	**3**	**2.68**	**1**	**3.13**	**0**	**0.00**	**1**	**2.86**	**1**	**5.89**
Cough	1	0.90	0	0.00	0	0.00	0	0.00	1	5.89
Interstitial lung disease	1	0.90	0	0.00	0	0.00	1	2.86	0	0.00
Rhinitis allergic	1	0.90	1	3.13	0	0.00	0	0.00	0	0.00
**Skin and subcutaneous tissue disorders**	**4**	**3.58**	**2**	**6.25**	**1**	**3.58**	**1**	**2.86**	**0**	**0.00**
Alopecia	2	1.79	1	3.13	1	3.58	0	0.00	0	0.00
Pruritus	1	0.90	1	3.13	0	0.00	0	0.00	0	0.00
Rash	1	0.90	0	0.00	0	0.00	1	2.86	0	0.00
**Surgical and medical procedures**	**13**	**11.61**	**5**	**15.63**	**3**	**10.72**	**4**	**11.43**	**1**	**5.89**
Eye laser surgery	3	2.68	3	9.38	0	0.00	0	0.00	0	0.00
Hysterectomy	1	0.90	0	0.00	1	3.58	0	0.00	0	0.00
Intraocular lens implant	1	0.90	0	0.00	0	0.00	1	2.86	0	0.00
Punctal plug insertion	8	7.15	2	6.25	2	7.15	3	8.58	1	5.89

**Vascular disorders**	**4**	**3.58**	**0**	**0.00**	**1**	**3.58**	**1**	**2.86**	**2**	**11.77**
Essential hypertension	1	0.90	0	0.00	0	0.00	0	0.00	1	5.89
Hypertension	3	2.68	0	0.00	1	3.58	1	2.86	1	5.89

Group 1: SA001 360 mg/day (180 mg twice daily); Group 2: SA001 720 mg/day (360 mg twice daily); Group 3: SA001 1,080 mg/day (540 mg twice daily); placebo. N = number of participants.

Supplementary Table S2. Exploratory Biomarkers (SSB (La), ANA, RF, IgG, IgA) Changes from Visit 2 (Baseline) to Visit 4 (Week 8) in Each Group

		Group 1		Group 2		Group 3		Placebo		
		N= 4		N= 3		N= 8		N= 6		p-value
**SSB(La)** (index)		**mean**	**SD**	**mean**	**SD**	**mean**	**SD**	**mean**	**SD**	
	Visit2	3.04	0.66	2.19	0.67	2.29	1.12	2.18	1.81	0.7256
	Visit4	2.97	0.77	2.35	0.89	2.17	1.14	2.09	1.65	0.7049
	Δ (Vist2-Vist4)	0.07	0.35	-0.16	0.33	0.12	0.55	0.09	0.25	0.8006
		**N**	**%**	**N**	**%**	**N**	**%**	**N**	**%**	
Visit2	positive	4	100.00	3	100.00	7	87.50	3	50.00	0.2127
	negative	0	0.00	0	0.00	1	12.50	3	50.00	
Visit4	positive	4	100.00	3	100.00	7	87.50	3	50.00	
	negative	0	0.00	0	0.00	1	12.50	3	50.00	0.2127

		Group 1		Group 2		Group 3		Placebo		p-value
		N= 4		N= 3		N= 8		N= 6		
**ANA**		**N**	**%**	**N**	**%**	**N**	**%**	**N**	**%**	
Visit2	positive	4	100.00	3	100.00	8	100.00	5	83.33	0.6190
	Weakly positive	0	0.00	0	0.00	0	0.00	1	16.67	
Visit4	positive	4	100.00	3	100.00	8	100.00	5	83.33	0.6190
	Weakly positive	0	0.00	0	0.00	0	0.00	1	16.67	

		Group 1		Group 2		Group 3		Placebo		
		N= 4		N= 3		N= 8		N= 6		p-value
**RF** (IU/ml)		**mean**	**SD**	**mean**	**SD**	**mean**	**SD**	**mean**	**SD**	
	Visit2	109.53	136.72	18.00	7.21	33.74	23.78	58.00	81.67	0.3444
	Visit4	105.53	131.09	19.97	2.57	32.61	24.22	58.82	82.05	0.3647
	Δ (Vist2-Vist4)	4.00	6.75	-1.97	6.37	1.13	1.87	-0.82	1.78	0.1931
**IgG** (mg/dl)		**mean**	**SD**	**mean**	**SD**	**mean**	**SD**	**mean**	**SD**	**p-value**
	Visit2	1958.50	484.44	1454.00	322.98	1760.25	450.89	1668.33	558.24	0.5762

	Visit4	1864.50	474.10	1437.33	247.51	1679.50	425.95	1789.67	722.21	0.7265
	Δ (Vist2-Vist4)	94.00	11.52	16.67	268.34	80.75	122.40	-121.33	185.16	0.1135
**IgA** (mg/dl)		**mean**	**SD**	**mean**	**SD**	**mean**	**SD**	**mean**	**SD**	**p-value**
	Visit2	345.25	226.96	287.33	150.49	270.13	92.96	300.67	151.33	0.8721
	Visit4	328.00	205.13	262.67	173.56	253.75	93.33	300.00	139.70	0.8291
	Δ (Vist2-Vist4)	17.25	23.77	24.67	56.66	16.38	19.97	0.67	34.38	0.6874

Group 1: SA001 360 mg/day (180 mg twice daily); Group 2: SA001 720 mg/day (360 mg twice daily); Group 3: SA001 1,080 mg/day (540 mg twice daily); placebo. N = number of participants.

Supplementary Table S3. Inclusion and exclusion criteria for SA001_04 Phase 2a

Inclusion criteria	Exclusion criteria
Primary Sjögren’s syndrome patients with dry eye and dry mouth symptoms who met all of the following inclusion criteria were eligible for enrollment in this study:<br>1) Adults aged between 19 and 80 years.<br>2) Diagnosed with primary Sjögren’s syndrome, with a total score of 4 or higher based on the following five criteria:<br>① Minor Salivary Gland Biopsy: Confirmation of lymphocytic sialadenitis with a focus score of ≥1 (1 focus/4mm²) (3 points).<br>※ Screening results may include test results obtained within six months prior to the consent date.<br>② Anti-SSA/Ro Antibody Positivity (3 points).<br>③ Ocular Staining Score (OSS): Score of ≥5 in at least one eye (1 point).<br>④ Schirmer’s Test (without anesthesia): Result of ≤5 mm/5 min in at least one eye (1 point).<br>⑤ Unstimulated Salivary Flow Rate Test: ≤0.1 ml/min (1 point).<br>3) SPEED (Standard Patient Evaluation of Eye Dryness) questionnaire score of ≥5 (0–4 Mild, 5–7 Moderate, 8+ Severe) for dry eye symptoms.<br>4) Provided voluntary written consent to participate in this clinical trial.	Patients to whom any of the following criteria applied were excluded from participation in this study:<br>1) Patients with secondary Sjögren’s syndrome.<br>2) Patients with severe blepharitis caused by Meibomian Gland Dysfunction (MGD).<br>3) Use of ophthalmic solutions that may affect efficacy evaluation (e.g., steroids, glaucoma medications, allergy treatments, antibiotics, ophthalmic gels, cyclosporine, diquafosol tetrasodium, etc.) within two weeks prior to the baseline visit (Visit 2).<br>4) Initiation or dose modification of systemic corticosteroids or immunosuppressants that may affect immune function within four weeks prior to the baseline visit (Visit 2).<br>5) Use of oral tetracycline or isotretinoin medications within four weeks prior to the baseline visit (Visit 2).<br>6) Initiation or dose modification of hydroxychloroquine within 12 weeks prior to the baseline visit (Visit 2).<br>7) Patients planning to undergo ocular surgery (including LASIK/LASEK) during the study period.<br>8) Patients requiring contact lens use during the study period.<br>9) History of ophthalmic surgery or ocular trauma within six months prior to the consent date.<br>10) Participation in another clinical trial involving drug or medical device administration within three months prior to the consent date.<br>11) Known hypersensitivity to the study drug components.<br>12) Patients with genetic disorders such as galactose intolerance, Lapp lactase deficiency, or glucose-galactose malabsorption.<br>13) Women with a positive pregnancy test (Serum hCG) at the screening visit (Visit 1) or those who do not agree to use at least one medically acceptable, effective contraceptive method* during the study.

	- Effective contraception methods include intrauterine devices (IUDs such as Loop or Mirena), double-barrier methods (diaphragm or condom/femidom + spermicide), and properly used non-oral contraceptives. Abstinence alone is not considered an acceptable method. Postmenopausal women (≥1 year) or those confirmed to be surgically sterile are exempt from this criterion.<br>14) Patients taking oral contraceptives during the clinical trial period.<br>15) Pregnant or breastfeeding women.<br>16) Patients with a history of drug or alcohol abuse.<br>17) Patients deemed unsuitable for study participation by the investigator.

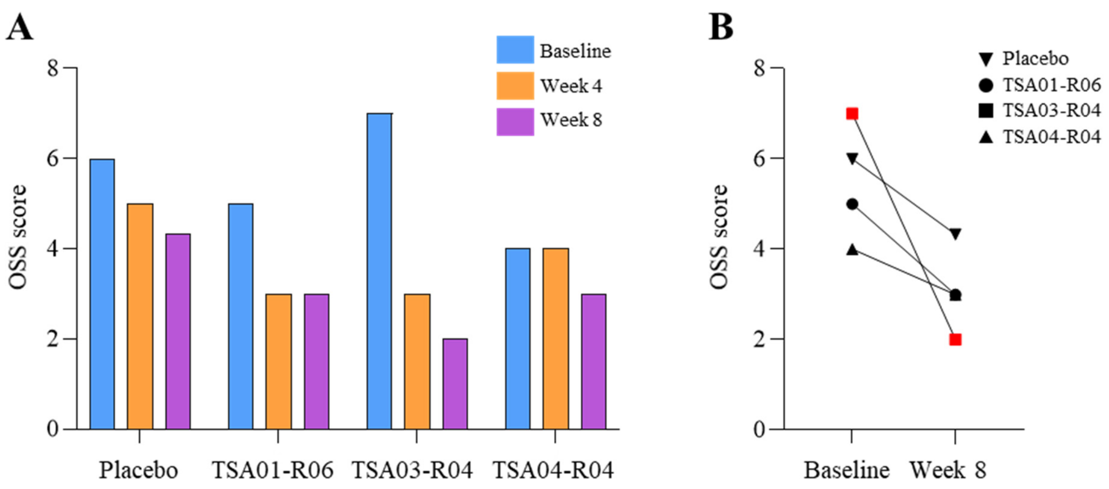

Figure S1. OSS Scores at Each Time Point and Change from Baseline to Week 8 in Treatment Group 2

(A) Individual OSS scores for each participant in Treatment Group 2 at baseline, Week 4, and Week 8, shown alongside the mean OSS values of the placebo group (n=6).

(B) Change in OSS score from baseline to Week 8 for each participant in Treatment Group 2, with the corresponding mean change in the placebo group presented for comparison.

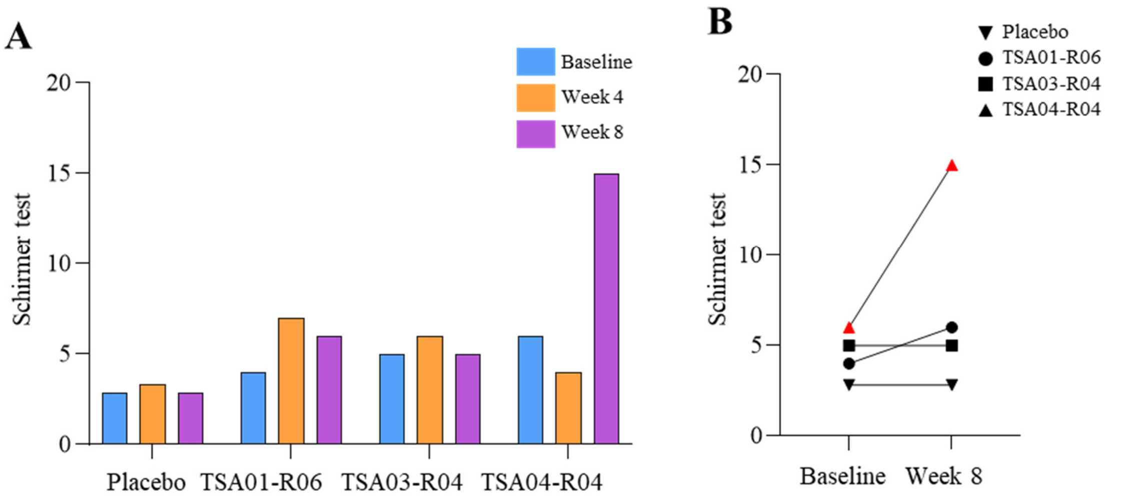

Figure S2. Shirmer test at Each Time Point and Change from Baseline to Week 8 in Treatment Group 2

(A) Individual Shirmer test for each participant in Treatment Group 2 at baseline, Week 4, and Week 8, shown alongside the mean Shirmer test values of the placebo group (n=6). (B) Change in Shirmer test from baseline to Week 8 for each participant in Treatment Group 2, with the corresponding mean change in the placebo group presented for comparison.

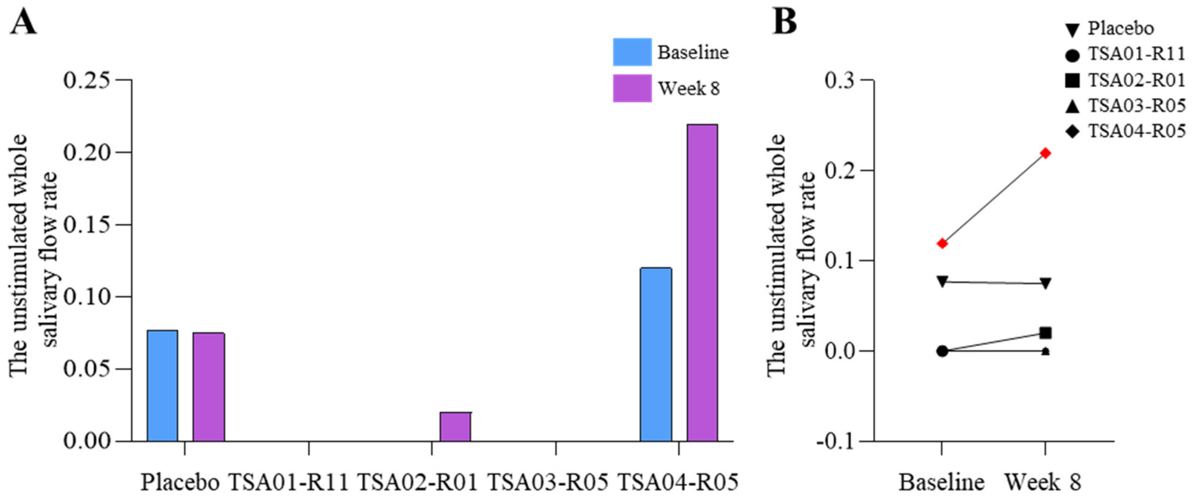

Figure S3. The unstimulated whole salivary flow rate at Each Time Point and Change from Baseline to Week 8 in Treatment Group 1

(A) Individual unstimulated whole salivary flow rate for each participant in Treatment Group 1 at baseline and Week 8, shown alongside the mean unstimulated whole salivary flow rate of the placebo group (n=6).

(B) Change in the unstimulated whole salivary flow rate from baseline to Week 8 for each participant in Treatment Group 1, with the corresponding mean change in the placebo group presented for comparison.
